# Wing-shaped walls: A directional effect of obstacles on manual avoidance

**DOI:** 10.1177/20416695241254959

**Published:** 2024-05-15

**Authors:** Yuki Harada, Hiroyuki Mitsudo

**Affiliations:** Faculty of Humanities, 12915 Kyoto University of Advanced Science, Kyoto city, Japan; Division of Psychology, Department of Human Sciences, Faculty of Human-Environment Studies, 12923 Kyushu University, Fukuoka, Japan

**Keywords:** manual movements, obstacle avoidance, attention, attention-movement interaction

## Abstract

Visual information can be used to plan, start, and coordinate manual movements in obstacle avoidance. An intriguing example of visuomotor coordination is the effect of wing-shaped walls, in which walls are oriented away from or toward a moving agent. A historical story from medieval Japan recounts that wing-shaped walls disrupted the agent's movement more when oriented toward the agent than when oriented away from the agent. This study aimed at examining whether the disruptive effect of wing-shaped walls occurs in a schematic situation represented on a 2D plane. In this study, we conducted psychophysical experiments in which participants were asked to move a stylus from a start point to a goal while avoiding multiple line obstacles that were arranged alternately at a course. In the two experiments, we manipulated the orientation and the size of the visible parts of the obstacles systematically. We found that the obstacles oriented toward the agent produced frequent contacts with the agent and attracted manual movements to the endpoints of obstacles. We discussed possible interpretations of the results in the context of attentional guidance.

When facing obstacles, people can visually recognize the characteristics of the obstacles and coordinate their own movements to avoid them. In a task where participants were asked to move a stylus from the start point to the goal, participants’ manual movements were coordinated in response to two obstacle lines moving in a certain direction (Aivar et al., 2008). Such visuomotor coordination has been found to be influenced by stimulus factors of obstacles such as a movement (Aivar et al., 2008), location (Brenner & Smeets, 2007), orientation (Sabes & Jordan, 1997), intrusion (Yamanaka & Stuerzlinger, 2020), and the distance from the agent (Chapman & Goodale, 2008). Moreover, it has been reported that the avoidance is influenced by the predictability of obstacles (van der Wel et al., 2007). For example, repeated presentations of obstacles produced a greater avoidance trajectory in manual movements even when the obstacles were not presented (Jax & Rosenbaum, 2007).

An intriguing example of the visuomotor coordination is “chidori-kake,” which means multiple wing-shaped walls (Figure 1) and appears in historical documents from medieval Japan. In this example, several walls were alternately arranged and oriented toward or away from a moving agent. In 1585, the wing-shaped walls were used in a local Japanese war between the Sanada and the Tokugawa troops (the first battle of Ueda: Digital Archive Portal in Ueda city, 2010). In this war, the wing-shaped walls were set up by defending soldiers (the Sanada troops, goal in Figure 1(a)) to lure the attacking soldiers (the Tokugawa troops, start in Figure 1(a)). Because the walls had been oriented away from the attacking soldiers, the soldiers could easily pass the wing-shaped walls. When the defending soldiers ambushed the attacking soldiers at the end of the course, the attackers could not easily retreat from the course (i.e., start to goal in Figure 1(b)), resulting in a successful defense. This episode suggests the effect of obstacle direction (slanting toward or away from the moving agent) on avoidance movements.

**Figure 1.**
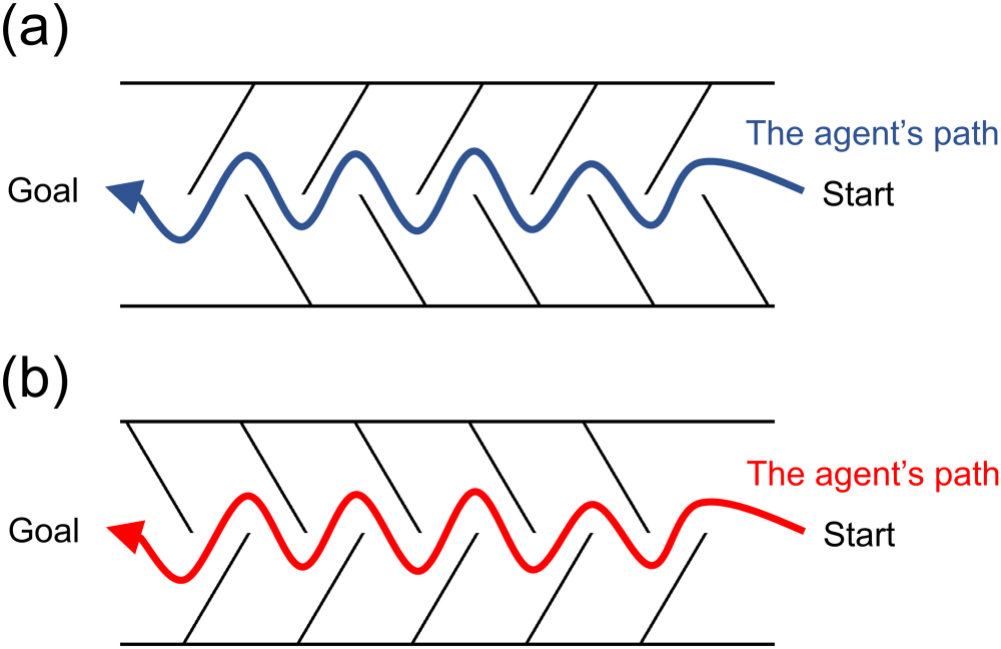
Bird's eye view of multiple wing-shaped walls (obstacles). (a) Walls oriented away from the moving agent. (b) Walls oriented toward the moving agent. According to the Digital Archive Portal in Ueda city (2010), the walls did not prevent soldiers from passing through the upper course, but prevented them from passing through the lower course.

Obstacles oriented toward people in real 3D space, such as the wing-shaped walls used in medieval Japan, can cause physical damage and disrupt their movement when contacted. A factor that we want to consider here is the perceptual and cognitive aspects of wing-shaped walls. Just as shown in the birds’ eye view of Figure 1(b), a person can visually recognize that the agent is unsafe when faced with a wall facing toward the agent. Therefore, such obstacles may be potentially threatening and thus emotionally salient. Given that emotional saliency captures viewers’ attention automatically (Mulckhuyse, 2018), the endpoint of the obstacles may capture attention. An important aspect is that visual attention can guide behaviors (Payne et al., 2017). Some studies have shown that saccade to a target occurs immediately before goal-directed movements occur (Johansson et al., 2001; Navarro et al., 2020). Welsh et al. (1999) found that when a target and a distractor were presented in the same scene, manual movements were attracted to the distractor location. The attraction to distractors has been observed when the saliency of distractors such as color (Moher et al., 2015) and luminance (Wood et al., 2011) was manipulated. Therefore, it can be predicted that the endpoints of wall-like obstacles oriented toward the agent induce contacts with the agent and attract manual movements to the endpoints. To our knowledge, no study has investigated this prediction.

This study examined the effect of obstacle direction on manual movements by using wing-shaped walls drawn on a 2D plane. In the two experiments reported here, participants were asked to move a stylus from the start to the goal positions while avoiding line segments that were arranged alternately. The orientation of obstacle lines was manipulated to systematically examine the directional effect of obstacles on manual movements. In addition, to examine whether and how the predictability for obstacles affects manual movements, the visibility of the obstacles was manipulated. The trajectory of manual movements, the contact between the stylus and the endpoint of obstacles, and movement times were analyzed.

## Experiment 1

The primary goal of Experiment 1 was to examine whether 2D wing-shaped walls affect manual movements. In addition to the orientation of obstacles, viewing condition was manipulated to investigate possible effects of predictability on manual movements. If the 2D wing-shaped walls affect manual movements irrespective of predictability, the obstacles oriented toward the agent would (a) increase the rate of contact with obstacles and movement times and (b) influence the trajectory.

### Method

#### Participants

Ten right-handed undergraduate and graduate students (two females) participated in the experiment. None was aware of the purpose of the experiment. Their age ranged from 21 to 26 years (*M *= 23.5, *SD *= 1.4). The participants had normal or corrected-to-normal visual acuity. The sample size was similar to that of an earlier study (Moher & Song, 2019). Written informed consent was obtained from all participants. The experiment was approved by the local ethics committee of the Faculty of Human-Environment Studies of Kyushu University (Approval number: 2016-015).

#### Apparatus

**Figure 2.**
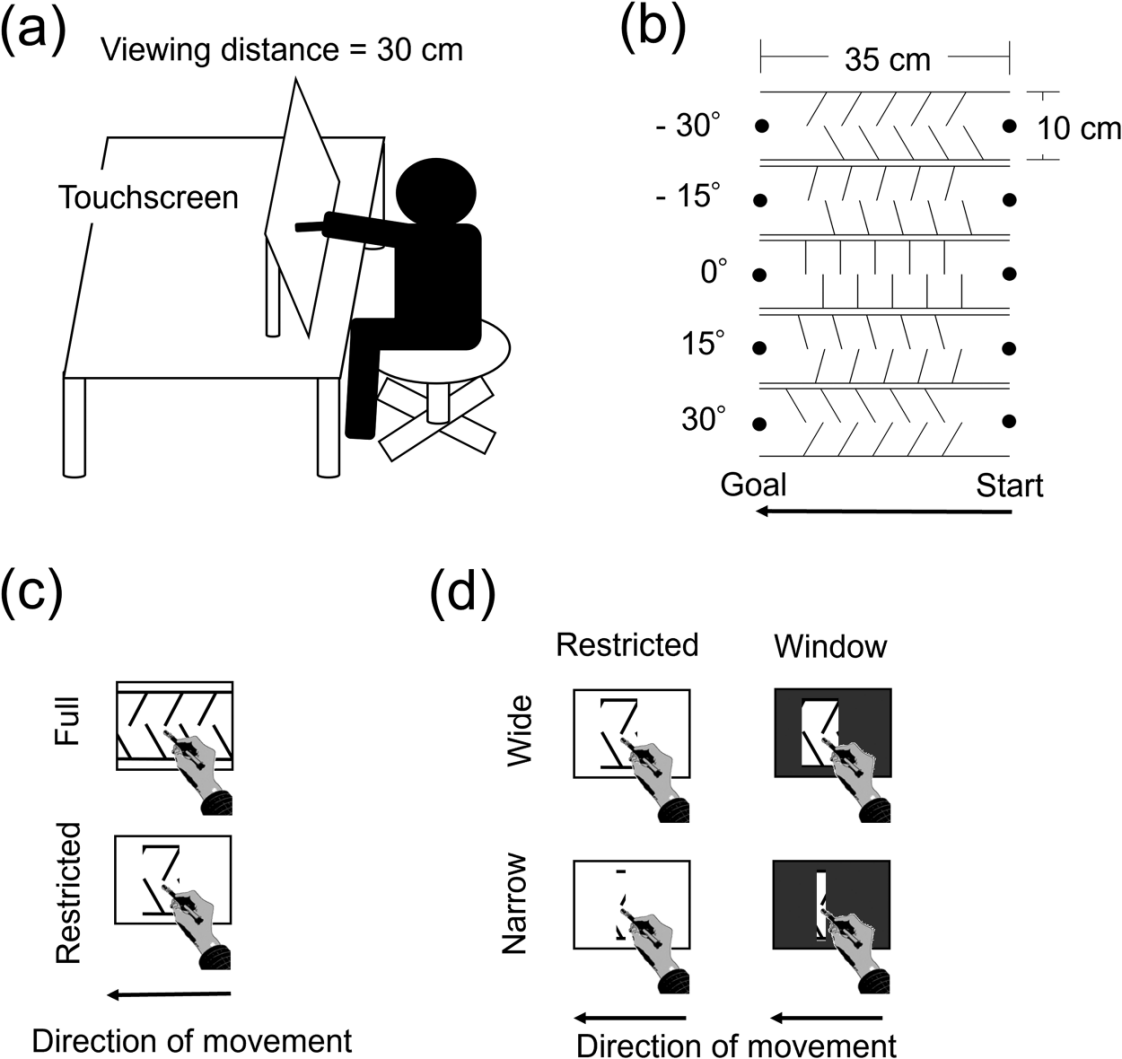
Schematic illustration of the settings in Experiments 1 and 2. (a) The experimental environment and apparatus. (b) The stimuli. The orientation of obstacles was varied at five levels in Experiment 1 and at three levels in Experiment 2. (c) The two viewing conditions in Experiment 1. (d) The four viewing conditions in Experiment 2.

A 23-inch touchscreen (DELL P2314T; 51 × 29 cm; 1920 × 1080 pixels; 60 Hz) was used to present stimuli and to record the trajectory of stylus movements (Figure 2(a)). The viewing distance was approximately 30 cm. The stimulus presentation and trajectory recording were controlled by MATLAB (Mathworks) with Psychtoolbox (Brainard, 1997; Kleiner et al., 2007; Pelli, 1997) running on a personal computer (DELL Inspiron 14z 5423). The stylus movement was sampled at 60 Hz.

#### Stimuli

Each course was composed of two horizontal lines (length, 35 cm), ten obstacle lines (height, 5 cm), and start and goal discs (diameter, 2 cm). The endpoints of obstacles along the virtual line from the start to the goal were located in the same position across different orientations. The lines and discs were drawn in black against a light grey background. As shown in Figure 2(b), the obstacles were oriented −30°, −15°, 0°, 15°, or 30° relative to the *y*-coordinate axis of the screen. The start disc was located on the right side of the course, and the goal disc was located on the left side.

The viewing condition (restricted, full) was manipulated to examine the effect of predictability on manual movements (Figure 2(c)). In the restricted viewing condition, the course was uniformly filled in light grey except for the 2-cm-wide area centered on the stylus. The mask moved according to the stylus movement. In the full viewing condition, no mask was presented so that the whole course was visible during a trial.

#### Procedure

In a silent and darkened room, the participants sat in a chair in front of the touchscreen and held the stylus with their right hand so as not to occlude the goal and the portion of the course to be tracked. At the beginning of each trial, when the participants touched the start disc, the disc color changed from black to white for 1,000 ms. If the participants moved the stylus outside the start disc at this time, the trial ended (false start trial). Immediately after the color of the disc returned to black, the participants were asked to move the stylus from the start to the goal while avoiding the obstacle lines. Speedy movements were required while maintaining accuracy. If the stylus was lifted from the touchscreen (lift trial) or contacted an obstacle line (error trial), the trial was aborted. By using the trial interruption procedure, we tried (a) to efficiently inform participants that the obstacles were dangerous for a trial completion and (b) to have participants perform the avoidance task seriously. When the stylus contacted the goal disc, the trial ended (successful trials).

Two independent variables were obstacle orientation and viewing condition. The obstacle orientation was manipulated within each experimental block, and the viewing condition was manipulated across blocks. The order of viewing conditions was counterbalanced across participants. The total number of experimental trials was 200, consisting of obstacle orientation (5) × viewing condition (2) × repetition (20). The number of repetitions was based on previous studies that measured movement trajectories, response times, and error rates (e.g., Aivar et al., 2015; Brenner & Smeets, 2007). The experiment lasted for approximately 1.5 h for each participant, including rests.

#### Analysis

Four dependent variables were the rate of contact around the obstacle endpoints, movement time, and two trajectory parameters of manual movements. The contact rates were calculated by the number of trials in which the stylus contacted an obstacle within a circle with a radius of 0.4 cm centered at each endpoint. For calculating the other dependent variables, we excluded data obtained from trials in which the stylus did not reach the goal (i.e., false start, lift, error trials). Excluded trials were 9.86% of total trials. The movement time was the time required to move the stylus from the start to the goal. To examine the effect of obstacle direction on the contacts and movement times in detail, we performed a curve-fitting analysis on trajectory data obtained from each successful trial. The sinusoidal function was fitted to the trajectory of manual movements using the following formula: *y *= a*sin (*xb *+ *c*) + *d*, where *x* is the *x*-coordinates of trajectories, *a* is the vertical amplitude of the trajectory (the *y* coordinates), *b* is the wavelength, *c* is the phase, *d* is the vertical offset. The horizontal range of curve-fitting was from 7 to 27 cm relative to the start. For each successful trial, the four free parameters were estimated by the least-squares method, and estimated values were averaged within each condition for each parameter of each participant.

For statistical significance tests, a within-participants analysis of variance (ANOVA) was performed on each dependent variable with the factors of obstacle orientation and viewing condition. For post-hoc multiple comparison tests, *p*-values were corrected with Holm's method. Analyses were performed using software R (version 4.1.2), R studio (version 2021.09.2. Build382), and Jeffreys's Amazing Statistics Program (version 0.16.3).

### Results and Discussion

#### Contact Rate and Movement Time

**Figure 3.**
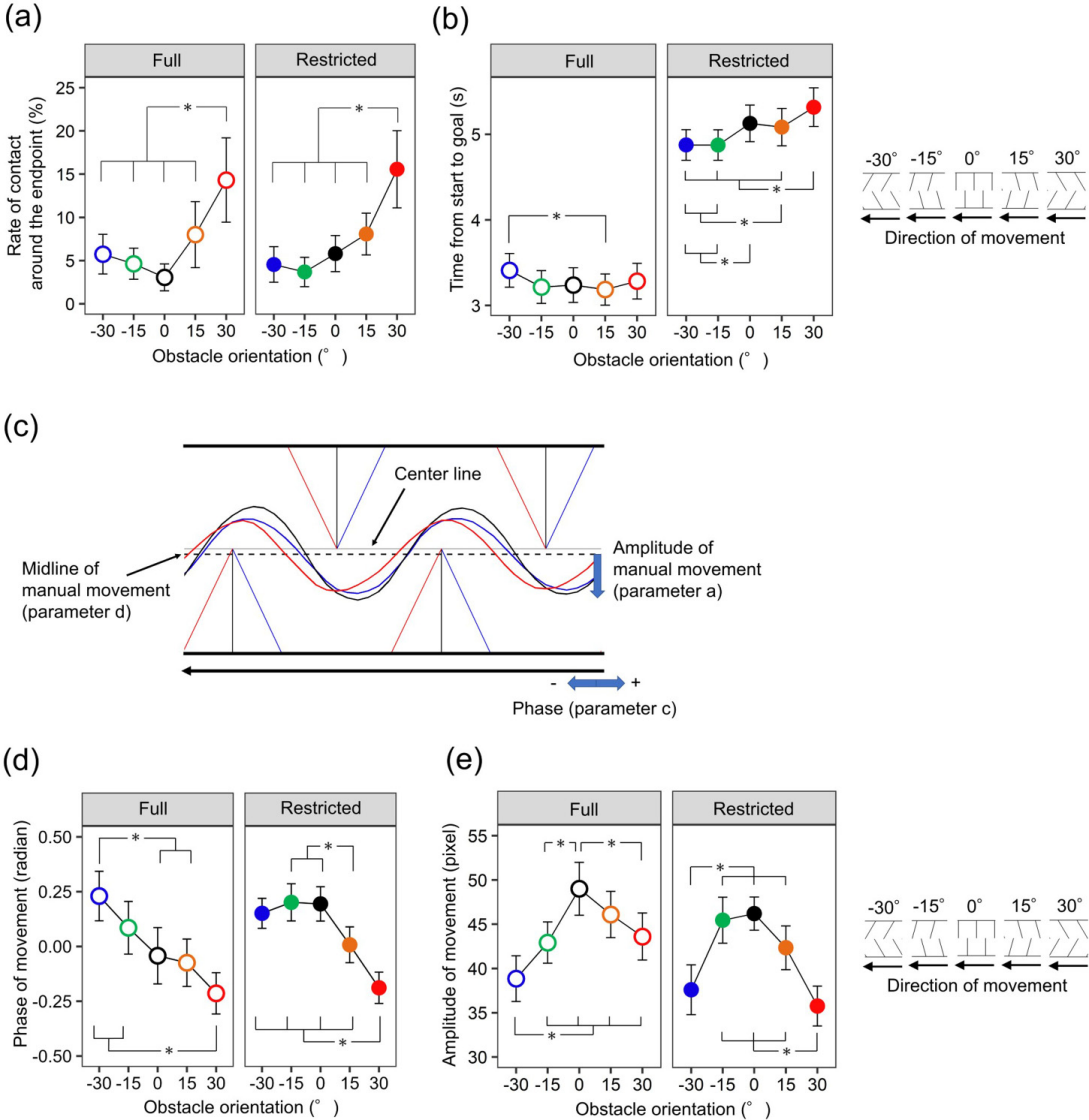
The results of Experiment 1. (a) The mean contact rates. (b) The mean movement times. (c) Mean trajectories of manual movements in the restricted condition. The red (−30°), black (0°), and blue (30°) lines and curves represent obstacles and averaged trajectories of manual movements, respectively. (d) The mean phase of sinusoidal curves fitted to the trajectory data. (e) The mean amplitude of sinusoidal curves fitted to the trajectory data. The error bars represent 95% confidence intervals. Asterisks represent significant differences (*p *< .05).

Figure 3(a) shows the rate of contact with endpoints averaged over the 10 participants. Contacts with the obstacle lines mostly occurred around the endpoints (91.6%). A two-way within-participants ANOVA with the factors of obstacle orientation and viewing condition was performed on the contact rates. The main effect of obstacle orientation was significant [*F* (4, 36) = 8.002, *p *< .001, *ηp*^2 ^= .471]. As shown by results of post-hoc tests (asterisks in Figure 3), irrespective of viewing condition, obstacles oriented toward the agent generally produced frequent contacts, consistent with the prediction. Neither the main effect of the viewing condition nor the two-way interaction was significant (*F*s < 0.201, *p*s > .881, *ηp*^2^s < .022). Therefore, our within-participants ANOVA demonstrates that the orientation-dependent disruptive effect was present in most participants.

Figure 3(b) shows the movement times averaged over the 10 participants. A two-way within-participants ANOVA was performed on the movement times. The main effects of obstacle orientation and viewing condition were significant [*F* (4, 36) = 8.106, *p *< .001, *ηp*^2 ^= .474; *F* (1, 9) = 73.028, *p *< .001, *ηp*^2 ^= .890, respectively]. The two-way interaction was also significant [*F* (4, 36) = 10.954, *p *< .001, *ηp*^2 ^= .549]. Results of post-hoc tests are shown by asterisks in Figure 3(b). These results suggest that obstacle orientation influenced the movement times, especially when the view was restricted.

#### Trajectory

Trajectory data were used to estimate the phase and amplitude of manual movements (Figure 3(c)). A negative phase value indicates a manual movement attracted to the endpoint, and the amplitude value indicates the size of vertical avoidance movements. Because the *R*^2^ value (Dosher et al., 2004) averaged across trials and participants was close to 1 (.906), these fittings are considered to be good.

Figure 3(d) shows the mean phase estimated by sinusoidal fitting. A two-way within-participants ANOVA was conducted on the phases. The main effect of obstacle orientation was significant [*F* (4, 36) = 28.403, *p *< .001, *ηp*^2 ^= .759] but the main effect of viewing condition was not [*F* (1, 9) = 1.473, *p *= .256, *ηp*^2 ^= .141]. The two-way interaction was also significant [*F* (4, 36) = 6.000, *p *< .001, *ηp*^2 ^= .400]. These results demonstrate that obstacles oriented toward the agent attracted manual movements to the endpoints.

Figure 3(e) shows the mean amplitude estimated by sinusoidal fitting. A two-way within-participants ANOVA was conducted on the amplitudes. The main effect of obstacle orientation and the two-way interaction were significant [*F* (4, 36) = 41.090, *p *< .001, *ηp*^2 ^= .820; *F* (4, 36) = 9.805, *p *< .001, *ηp*^2 ^= .521, respectively], but the main effect of viewing condition was not [*F* (1, 9) = 1.371, *p *= .272, *ηp*^2 ^= .132]. These results suggest that obstacles arranged vertically on the course produced larger avoidance movements, and these patterns were different from those of the phase analysis.

To summarize, the obstacles oriented toward the agent produced more frequent contacts with the obstacle endpoints and longer movement times. The pattern of results is largely consistent with those of the phase, but not of the amplitude of manual movements.

## Experiment 2

The purpose of Experiment 2 was to replicate the effect of obstacle direction on the manual movements in different stimulus conditions. The restricted view condition of Experiment 1 had a somewhat unnatural appearance since the obstacles appeared suddenly in blank space. Therefore, we compared manual movements between conditions where a part of the obstacles appeared through an invisible aperture (as in Experiment 1) and where the aperture that gave rise to an impression of “window” was used (Figure 2(d)). To further examine the effect of predictability, the width of view was also independently manipulated. If the effect of obstacle direction on manual movements was robust, the orientation would influence both the rate of contact with the endpoints and the phase of movements in these conditions.

### Method

The methods were the same as those of Experiment 1 except for the following.

#### Participants

Eleven right-handed undergraduate and graduate students (six females) participated in the experiment. Their ages ranged from 21 to 27 years (*M *= 23.9, *SD *= 1.7). Four of them also participated in Experiment 1.

#### Procedure and Analysis

Independent variables were obstacle orientation (−30, 0, 30°), the width of view (wide, narrow), and the type of view (restricted, window). Their illustrations are shown in Figure 2(d). The narrow view masked the course outside the 0.4-cm-wide area centered at the current stylus *x* position, and the wide view masked the course outside the 2-cm-wide area centered at the current stylus *x* position as in Experiment 1. In the window viewing condition, the masking region was drawn in dark grey so as to simulate a situation in which participants viewed the course through an aperture. In the restricted viewing condition, the masking region was drawn in light grey (the same as in Experiment 1). A three-way within-participants ANOVA was performed with factors of obstacle orientation, view width, and view type.

### Results and Discussion

#### Contact Rate and Movement Time

**Figure 4.**
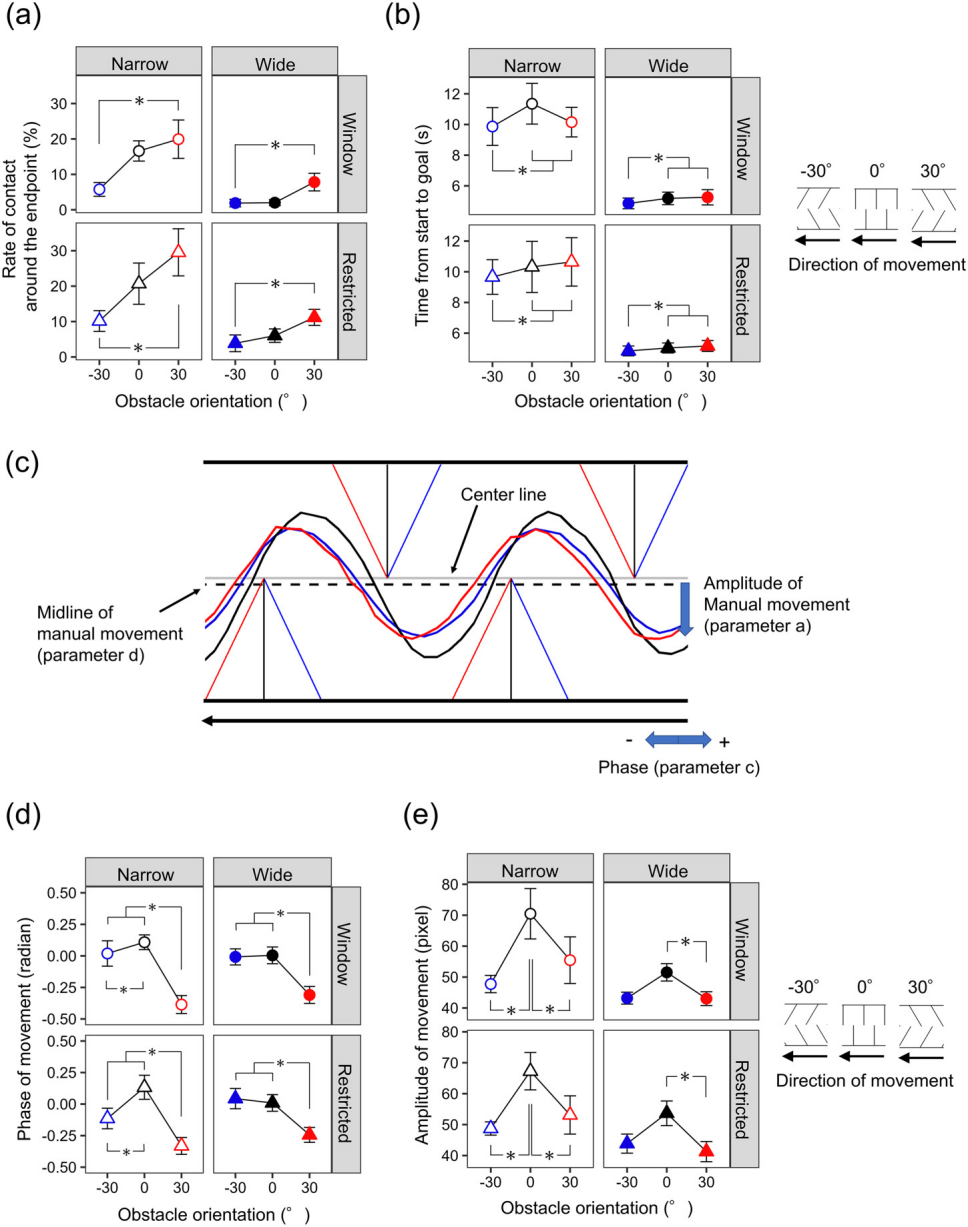
The results of Experiment 2. (a) The mean contact rates. (b) The mean movement times. (c) Mean trajectories of manual movements in the narrow window condition. The red (−30°), black (0°), and blue (30°) lines and curves represent obstacles and averaged trajectories of manual movements, respectively. (d) The mean phase of sinusoidal curves fitted to the trajectory data. (e) The mean amplitude of sinusoidal curves fitted to the trajectory data. The error bars represent 95% confidence intervals. Asterisks represent significant differences (*p *< .05).

Figure 4(a) shows the mean rate of contact with the endpoints. Many contacts with the obstacle lines occurred around the endpoints (76.0%). A three-way within-participants ANOVA with the factors of obstacle orientation, view width, and view type was performed on the contact rates. The main effects of obstacle orientation, view width, and view type were significant [*F* (2, 20) = 9.487, *p *= .001, *ηp*^2 ^= .487; *F* (1, 10) = 31.310, *p *< .001, *ηp*^2 ^= .758; *F* (1, 10) = 7.537 *p *= .021, *ηp*^2 ^= .430, respectively]. The results are generally consistent with those of Experiment 1: Irrespective of view width or type, obstacles oriented toward the agent produced frequent contacts. The interactions were not significant (*F*s < 3.244, *p*s > .060, *ηp*^2^s < .245).

Figure 4(b) shows the mean movement times. A three-way within-participants ANOVA was performed on the movement times. The main effects of obstacle orientation and view width were significant [*F* (2, 20) = 6.777, *p *= .006, *ηp*^2 ^= .404; *F* (1, 10) = 34.634, *p *< .001, *ηp*^2 ^= .776, respectively], but the main effect of view type was not [*F* (1, 10) = 0.111, *p *= .746, *ηp*^2 ^= .011]. No interaction was significant (*F*s < 3.133, *p*s > .065, *ηp*^2^s < .239). These results suggest that obstacles slanted away from the agent facilitated manual movements, and they are consistent with those obtained in the restricted viewing condition of Experiment 1.

#### Trajectory

As in Experiment 1, the trajectory of manual movements was used to estimate the phase and amplitude parameters in sinusoidal fitting (Figure 4(c)). Because the mean *R*^2^ value was close to 1 (.896), these fittings are considered to be good. Figure 4(d) shows the mean phase of movements. A three-way within-participants ANOVA was performed on the phases. The main effect of obstacle orientation was significant [*F* (2, 20) = 40.664, *p *< .001, *ηp*^2 ^= .803], but the other main effects were not (*F*s < 0.085, *p*s > .777, *ηp*^2^s < .008). The two-way interaction between obstacle orientation and view width was significant [*F* (2, 20) = 4.031, *p *= .034, *ηp*^2 ^= .287]. These results showed that obstacles oriented toward the agent attract manual movements to the endpoints.

Figure 4(e) shows the mean amplitudes of manual movements. A three-way within-participants ANOVA was performed on the amplitudes. The main effect of obstacle orientation was significant [*F* (2, 20) = 20.248, *p *< .001, *ηp*^2 ^= .669], but the other main effects were not (*F*s < 3.839, *p*s > .079, *ηp*^2^s < .277). The two-way interaction between view width and obstacle orientation was significant [*F* (2, 20) = 4.811, *p *= .020, *ηp*^2 ^= .325]. The other interactions were not significant (*F*s < 0.684, *p*s > .516, *ηp*^2^s < .064). These results showed that obstacles arranged orthogonally to the overall movement direction produced greater avoidance movements. To summarize, Experiment 2 generally replicated the results of Experiment 1.

## General Discussion

This study examined the effect of obstacle direction on manual movements. The participants were asked to move the stylus from the start to the goal while avoiding obstacles with various orientations. We found that obstacles oriented toward the moving agent produced frequent contacts with the obstacle endpoints and slowed down manual movements in many viewing conditions. The same obstacles oriented toward the agent also shifted the phase of manual movement trajectories toward the obstacles. These results can be explained by the idea that the “oriented-toward-the-agent” obstacles attracted manual movements to the endpoints.

**Figure 5.**
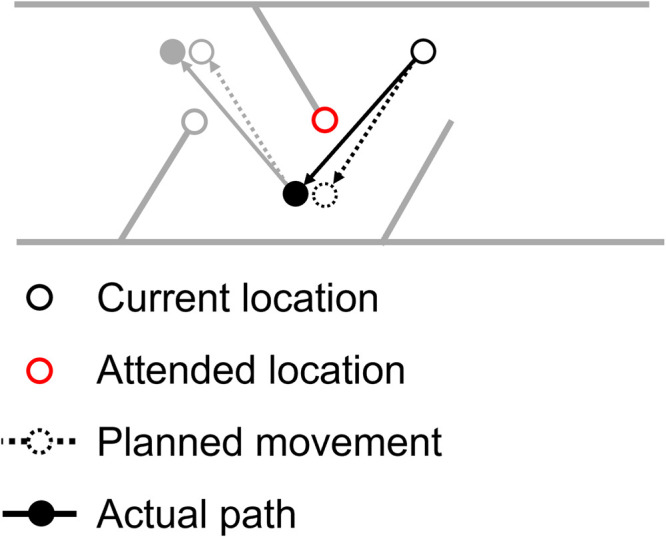
Schematic illustration of the averaging account. The endpoint of an obstacle is assumed to capture attention, producing an attraction of manual movements to the endpoint. As a result, the actual movement becomes closer to the averaged trajectory between the endpoint and the planned path.

Does attention explain the present results? As indicated by the narrative referred in the introduction, when contacted, obstacles oriented toward the agent in real 3D space would cause physical damage and pain more strongly than those with different orientations. We presume that the oriented-toward-the-agent obstacles are threatening even when schematically drawn on a 2D plane. Threatening stimuli promote attentional selection at the early stage of visual processing (Mulckhuyse, 2018; Öhman et al., 2001). Given that attention guides manual movements (Moher et al., 2015; Wood et al., 2011), attention would be attracted by the oriented-toward-the-agent obstacles, resulting in trajectories averaged between the planned movement target and the attended location (Figure 5). This idea can explain the higher contact rates in the narrow view condition of Experiment 2, since the endpoints of oriented-toward-the-agent obstacles more suddenly appeared around the agent than those in the other conditions.

The averaging account presumes that threatening stimuli promote approaching behaviors. This may sound strange or counter-intuitive because defense systems are generally motivated to escape from threatening objects (LeDoux & Daw, 2018). Mixed evidence has been reported for the effect of threat on approach-avoidance behaviors: promoting avoidance behaviors (Lebowitz et al., 2015; Rinck et al., 2021) and promoting approaching behaviors (Zsido et al., 2023). Zsido et al. observed that individuals with snake phobia moved a mouse cursor to the image of snakes more quickly than to that of neutral pictures. One reason for the counter-intuitive behaviors could be that responses to threatening stimuli are composed of not only passive avoidance (i.e., simply escaping from threatening stimuli) but also active coping (Carver & Harmon-Jones, 2009), in which individuals temporarily approach the stimuli to judge the risk, to calculate an escape route, and to prepare a defense. Therefore, we speculate that approaching behaviors to the oriented-toward-the-agent stimuli may be a by-product of adaptive ones that are useful in some other situations.

In the tentative averaging account, we do not explicitly presume that an attraction of manual movements to an endpoint is mediated by a conscious, perceived distortion of visual space. Suzuki and Cavanagh (1997) and Yamada et al. (2011) reported that transient attention expands perceived visual space around the attended location. As a result, our participants might try to counteract the expanded visual space around the endpoints of oriented-toward-the-agent obstacles by moving the stylus near the endpoints. The space distortion scheme is also consistent with the present results. Note that the averaging account and the space distortion account are not mutually exclusive.

The effect of obstacle direction on the contact rates might be explained by the predictability of obstacles. Especially in restricted viewing, the obstacles oriented away from the agent might act as cues to predict the location of obstacle endpoints since they appear from two horizontal bounding lines to the endpoints around the moving path. This explanation is consistent with the results of Experiment 2, in that the contact rates were lower for the obstacles oriented away from the agent than for the other obstacles. However, this idea cannot explain the difference in the contact rates between the oriented-toward-the-agent and vertical obstacles. In these conditions, the obstacle endpoints cannot be predicted from the remaining part of the lines since the endpoints appeared before the remaining part in the oriented-toward-the-agent obstacles and a whole line appeared at the same time in the vertical obstacles. Nevertheless, the contact rates were higher for the oriented-toward-the-agent obstacles than for the vertical obstacles. Therefore, the effect of obstacle direction on the contact rates cannot be fully explained by the predictability of the obstacles.

An effect of view size on the dependent measures was found in both experiments. In Experiment 1, overall movement times were much shorter in the full view condition than in the restricted view condition; trajectory phases changed gradually only in the full view condition. In Experiment 2, overall movement times were much shorter in the wide view condition than in the narrow view condition. One interpretation is that this view size effect results from anticipatory attentional control in response to multiple obstacles. Baldauf and Deubel (2008) have asserted that before the execution of manual movements, attention can be divided among multiple visible objects such as the goal, distractors, and obstacles. The attentional landscape has been suggested to coordinate the planning and action of manual movements (Baldauf, 2018). This idea can explain the view size effect since in the wider viewing conditions, participants can know not only the position of obstacles located near the stylus but also that of obstacles located far from the stylus in advance. Such a viewing condition may construct an attentional landscape for efficient manual movements.

An anonymous reviewer asked us to consider the possibility that the orientation-dependent disruption changed over time. To investigate this issue, we divided entire trials (200 and 240 trials in Experiments 1 and 2, respectively) into four equally sized successive bins and calculated the contact rate in each trial bin (viewing conditions were collapsed). A two-way within-participants ANOVA was conducted on the contact rates with factors of obstacle orientation and trial bin for each experiment. In Experiments 1 and 2, the main effects of obstacle orientation were significant (*F*s > 7.409, *p*s < .001, *ηp*^2^s > .452), but neither the main effect of trial bin nor the interaction was significant (*F*s < 1.025, *p*s > .397, *ηp*^2^s < .102). Therefore, the orientation-dependent disruptive effect persisted over time and was unaffected by practice during the experiment.

The present study demonstrated that wing-shaped walls have a distracting effect on avoidance movements when drawn as a 2D schematic representation. This confirms the narrative described in the first battle of Ueda in medieval Japan (Digital Archive Portal in Ueda city, 2010) in terms of the perceptual and cognitive aspects. That is, the appreciation of the walls oriented toward the agent would be threatening and thus attention-demanding even when the agent did not physically contact the walls. From a theoretical perspective, it will be interesting to examine whether the present results can be simulated by kinematic modeling (Verheij et al., 2012) originally developed for digit movements for grasping. From a practical perspective, along with Yamanaka and Stuerzlinger (2020), the previous and present findings on visuomotor coordination will be helpful in improving human–computer interfaces where the agent's cursor movement is required, such as when viewing web pages or playing computer games.

Taken together, the results of this study provide empirical evidence for the effect of obstacle direction on manual movements. Our results demonstrated that obstacles oriented toward the agent produced frequent contacts and attracted manual movements to the obstacle endpoints. We discussed possible interpretations of the present results in the context of attentional guidance. To examine this issue further, it will be useful to measure eye movements or attended locations during the chidori-kake task.
